# Multidrug resistance, inappropriate empiric therapy, and hospital mortality in *Acinetobacter baumannii* pneumonia and sepsis

**DOI:** 10.1186/s13054-016-1392-4

**Published:** 2016-07-11

**Authors:** Marya D. Zilberberg, Brian H. Nathanson, Kate Sulham, Weihong Fan, Andrew F. Shorr

**Affiliations:** EviMed Research Group, LLC, PO Box 303, Goshen, MA 01032 USA; OptiStatim, LLC, Longmeadow, MA USA; The Medicines Company, Parsippany, NJ USA; Washington Hospital Center, 110 Irving Street NW, Washington, DC 20010 USA

**Keywords:** Pneumonia, Sepsis, *Acinetobacter baumannii*, Multidrug resistance, Inappropriate empiric therapy, Outcomes

## Abstract

**Background:**

The relationship between multidrug resistance (MDR), inappropriate empiric therapy (IET), and mortality among patients with *Acinetobacter baumannii* (AB) remains unclear. We examined it using a large U.S. database.

**Methods:**

We conducted a retrospective cohort study using the Premier Research database (2009–2013) of 175 U.S. hospitals. We included all adult patients admitted with pneumonia or sepsis as their principal diagnosis, or as a secondary diagnosis in the setting of respiratory failure, along with antibiotic administration within 2 days of admission. Only culture-confirmed infections were included. Resistance to at least three classes of antibiotics defined multidrug-resistant AB (MDR-AB). We used logistic regression to compute the adjusted relative risk ratio (RRR) of patients with MDR-AB receiving IET and IET’s impact on mortality.

**Results:**

Among 1423 patients with AB infection, 1171 (82.3 %) had MDR-AB. Those with MDR-AB were older (63.7 ± 15.4 vs. 61.0 ± 16.9 years, *p* = 0.014). Although chronic disease burden did not differ between groups, the MDR-AB group had higher illness severity than those in the non-MDR-AB group (intensive care unit 68.0 % vs. 59.5 %, *p* < 0.001; mechanical ventilation 56.2 % vs. 42.1 %, *p* < 0.001). Patients with MDR-AB were more likely to receive IET than those in the non-MDR-AB group (76.2 % MDR-AB vs. 13.8 % non-MDR-AB, *p* < 0.001). In a regression model, MDR-AB strongly predicted receipt of IET (adjusted RRR 5.5, 95 % CI 4.0–7.7, *p* < 0.001). IET exposure was associated with higher hospital mortality (adjusted RRR 1.8, 95 % CI 1.4–2.3, *p* < 0.001).

**Conclusions:**

In this large U.S. database, the prevalence of MDR-AB among patients with AB infection was > 80 %. Harboring MDR-AB increased the risk of receiving IET more than fivefold, and IET nearly doubled hospital mortality.

## Background

The Centers for Disease Control and Prevention considers *Acinetobacter baumannii* (AB) a “serious” threat [1]. AB’s resistance mechanisms target both first-line and salvage broad-spectrum agents, with approximate doubling in carbapenem and multidrug resistance (MDR) in the United States over the last decade [2, 3]. In addition to its public health implications, the rising tide of drug resistance presents a difficult clinical conundrum. In serious infections, appropriate initial therapy determines clinical outcomes. However, more extensive drug resistance makes it a challenge to select appropriate treatment [4–13]. Carbapenem resistance among AB in severe sepsis and/or septic shock increases the risk of receiving inappropriate empiric therapy (IET) nearly threefold, raising the risk of death [14]. Unfortunately, using carbapenems as empiric therapy in hopes of minimizing IET drives increasing carbapenem resistance. Because of the limited data on this issue in AB, we conducted a multicenter, retrospective cohort study to explore the impact of MDR in IET and of IET on hospital mortality in AB.

## Methods

We conducted a multicenter retrospective cohort study of patients admitted to the hospital with pneumonia and/or sepsis and included in the Premier Research database in the 2009–2013. We hypothesized that multidrug-resistant AB (MDR-AB) (primary exposure) increases the risk of receiving IET (primary outcome), and that IET increases hospital mortality. Because this study used already-existing, Health Insurance Portability and Accountability Act (“HIPAA”)-compliant, fully de-identified data, it was exempt from institutional review board (IRB) review.

### Patient population

Patients were included if they were adults (aged ≥ 18 years) hospitalized with pneumonia and/or sepsis. Pneumonia was identified by the principal diagnosis International Classification of Diseases, Ninth Revision, Clinical Modification (ICD-9-CM) codes 481–486, or by respiratory failure codes (518.81 or 518.84) with pneumonia as a secondary diagnosis. Sepsis was identified by the principal diagnosis codes 038, 038.9, 020.0, 790.7, 995.92, or 785.52, or by respiratory failure codes (518.81 or 518.84) with sepsis as a secondary diagnosis [15–18]. Only patients with community-onset (present on admission) infection and antibiotic treatment beginning within the first 2 hospital days and continued for at least three consecutive days or until discharge were included [15–17]. Patients were excluded if they had been transferred from another acute care facility, had cystic fibrosis, or had a hospital length of stay of 1 day or less. Those with both pneumonia and sepsis were included in the pneumonia group. Patients were followed until death in or discharge from the hospital. Only patients with a positive AB culture from a pulmonary or blood source who met the above criteria were included in the analysis.

### Data source

The Premier Research database, an electronic laboratory, pharmacy, and billing data repository for 2009–2013, contains approximately 15 % of all U.S. hospitalizations nationwide. In addition to patient age, sex, race and/or ethnicity, principal and secondary diagnoses, and procedures, the database contains a date-stamped log of all medications, laboratory tests, and diagnostic and therapeutic services charged to the patient or the patient’s insurer. We used data from 176 U.S. institutions that submit microbiological data into the database. Eligible time began only following the commencement of microbiological data submission by each institution.

### Baseline variables

We classified infection (pneumonia or sepsis) as healthcare-associated (HCA) if one or more of the following were present: (1) prior hospitalization within 90 days of the index hospitalization, (2) hemodialysis, (3) admission from a long-term care facility, and/or (4) immune suppression. All other infections were considered community-acquired (CA). Patient-level factors included demographic variables and comorbid conditions. Charlson comorbidity index score was computed as a measure of the burden of chronic illness, while intensive care unit (ICU) admission, mechanical ventilation, and vasopressor use served as markers for disease severity. Hospital-level characteristics examined were geographic region, size, teaching status, and urbanicity.

### Microbiological and treatment-related variables and definitions

Blood and respiratory cultures had to be obtained within the first 2 days of hospitalization. AB isolates were classified as S (susceptible), I (intermediate), or R (resistant). For the purposes of the present analyses, I and R were grouped together as nonsusceptible. MDR-AB was defined, per Magiorakos et al., as any AB resistant to at least one agent in at least three antimicrobial classes [19]. Similarly, extensively drug resistant AB (XDR-AB) was defined as an AB resistant to at least one agent in all but two or fewer classes listed above, and pandrug-resistant AB (PDR-AB) as an AB resistant to all antimicrobial agents listed above [19].

IET was present if the antibiotic administered did not cover the organism or if coverage did not start within 2 days of obtaining the positive culture. Because the role of combination therapy in treating AB is not well defined, combination therapy was not included in the definition of IET [20]. IET was deemed “indeterminate” if the susceptibility of AB to the regimen received was not reported. These cases were excluded from the IET analysis. All microbiological testing was performed at the institutions contributing data to the database and conformed to the Clinical & Laboratory Standards Institute standards.

### Statistical analyses

We compared characteristics of patients infected with MDR-AB with those of patients with non-MDR-AB infection, as well as characteristics of patients who received IET with those of patients treated with non-IET. Continuous variables were reported as means with SD when distributed normally or as medians with 25th and 75th percentiles when skewed. Differences between mean values were tested via Student’s *t* test, and differences between medians were assessed using the Mann-Whitney *U* test. Categorical data were summarized as proportions, and chi-square test or Fisher’s exact test (when cell counts were ≤4) was used to examine differences between groups.

We developed a generalized logistic regression model to explore the relationship between MDR-AB and the risk of IET. Covariates in the model included demographics (sex, age, whether the infection was HCA), Elixhauser comorbidities, and measures of illness severity by hospital day 2. We calculated the relative risk ratio with 95 % CI of receiving IET for MDR-AB vs. non-MDR-AB, based on Huber-White robust standard errors clustered at the hospital level [21]. To confirm our results, we created two other models: (1) a nonparse model that included all of the predictors in the generalized logistic regression model with a large number of additional treatments present or absent by hospital day 2, and (2) a propensity-matched model with propensity for MDR-AB derived from a logistic regression model using the nonparse model’s predictors, and MDR-AB matched to non-MDR-AB patients using a 5:1 Greedy algorithm [22, 23].

All tests were two-tailed, and a *p* value < 0.05 was deemed a priori to represent statistical significance. All analyses were performed in Stata/MP 13.1 for Windows software (StataCorp LP, College Station, TX, USA).

## Results

Among the 229,028 enrolled patients with pneumonia or sepsis, 1423 (0.6 %) had a pulmonary or blood culture positive for AB, of which 1171 (82.3 %) were MDR, 239 (16.8 %) were XDR, and 0 (0.0 %) were PDR. Patients with MDR-AB were older (63.7 ± 15.4 vs. 61.0 ± 16.9 years, *p* = 0.014) than those with non-MDR-AB, while the racial distributions were comparable in both groups (Table 1). Although the distribution of some chronic conditions varied, there was no difference between the groups in the Charlson comorbidity index (Table 1). MDR-AB was more common than non-MDR-AB in the West and the Midwest, in urban hospitals, and in hospitals of medium size (200–499 beds). Both large hospitals (500+ beds) and those with an academic program were less likely to have MDR-AB than non-MDR-AB (Table 1).

**Table 1:** Baseline characteristics

	Non-MDR-AB (*n* = 252)	%	MDR-AB (*n* = 1171)	%	*p* Value
Mean age, years (SD)	61.0 (16.9)		63.7 (15.4)		0.014
Male sex	134	53.2 %	633	54.1 %	0.799
Race/ethnicity					
White	134	53.2 %	633	54.1 %	< 0.001
Black					
Hispanic	159	63.1 %	738	63.0 %	
Other	55	21.8 %	276	23.6 %	
Admission source					
Non-healthcare facility (including from home)	167	66.3 %	573	48.9 %	< 0.001
Clinic	14	5.6 %	26	2.2 %	
Transfer from ECF	13	5.2 %	280	23.9 %	
Transfer from another non-acute care facility	3	1.2 %	45	3.8 %	
Emergency department	54	21.4 %	236	20.2 %	
Other	1	0.4 %	11	1.0 %	
Elixhauser comorbidities					
Congestive heart failure	61	24.2 %	353	30.1 %	0.060
Valvular disease	21	8.3 %	92	7.9 %	0.800
Pulmonary circulation disease	16	6.3 %	107	9.1 %	0.153
Peripheral vascular disease	33	13.1 %	145	12.4 %	0.756
Paralysis	32	12.7 %	292	24.9 %	<0.001
Other neurological disorders	44	17.5 %	300	25.6 %	0.006
Chronic pulmonary disease	108	42.9 %	507	43.3 %	0.898
Diabetes without chronic complications	65	25.8 %	390	33.3 %	0.020
Diabetes with chronic complications	21	8.3 %	96	8.2 %	0.943
Hypothyroidism	28	11.1 %	182	15.5 %	0.072
Renal failure	66	26.2 %	359	30.7 %	0.160
Liver disease	17	6.7 %	37	3.2 %	0.007
Peptic ulcer disease with bleeding	0	0.0 %	0	0.0 %	1.000
AIDS	0	0.0 %	0	0.0 %	1.000
Lymphoma	1	0.4 %	16	1.4 %	0.336
Metastatic cancer	20	7.9 %	30	2.6 %	< 0.001
Solid tumor without metastasis	17	6.7 %	31	2.6 %	0.001
Rheumatoid arthritis/collagen vascular	5	2.0 %	46	3.9 %	0.132
Coagulopathy	45	17.9 %	134	11.4 %	0.005
Obesity	41	16.3 %	191	16.3 %	0.987
Weight loss	49	19.4 %	392	33.5 %	< 0.001
Fluid and electrolyte disorders	145	57.5 %	628	53.6 %	0.258
Chronic blood loss anemia	5	2.0 %	16	1.4 %	0.461
Deficiency anemia	97	38.5 %	593	50.6 %	< 0.001
Alcohol abuse	22	8.7 %	35	3.0 %	< 0.001
Drug abuse	16	6.3 %	29	2.5 %	0.001
Psychosis	13	5.2 %	77	6.6 %	0.402
Depression	29	11.5 %	161	13.7 %	0.343
Hypertension	158	62.7 %	669	57.1 %	0.104
Charlson comorbidity index score					
0	58	23.0 %	247	21.1 %	0.542
1	60	23.8 %	298	25.4 %	
2	50	19.8 %	244	20.8 %	
3	35	13.9 %	179	15.3 %	
4	21	8.3 %	112	9.6 %	
5+	28	11.1 %	91	7.8 %	
Mean (SD)	2.2 (2.4)		2.0 (1.9)		0.096
Median [IQR]	2 [1–3]		2 [1–3]		0.873
Hospital characteristics					
U.S. census region					
Midwest	49	19.4 %	377	32.2 %	< 0.001
Northeast	54	21.4 %	164	14.0 %	
South	122	48.4 %	436	37.2 %	
West	27	10.7 %	194	16.6 %	
Number of beds					
< 200	26	10.3 %	140	12.0 %	0.007
200–299	49	19.4 %	272	23.2 %	
300–499	84	33.3 %	454	38.8 %	
500+	93	36.9 %	305	26.0 %	
Teaching	137	54.4 %	537	45.9 %	0.014
Urban	233	92.5 %	1135	96.9 %	0.001

*MDR-AB* multidrug-resistant *Acinetobacter baumannii*, *ECF* extended care facility

**Fig. 1:**
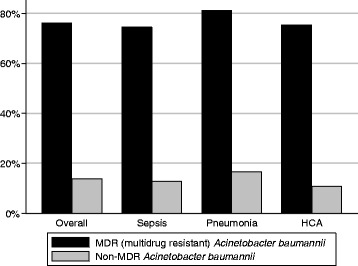
Inappropriate empiric therapy as a function of multidrug resistance (MDR). *HCA* healthcare-associated

In both groups (MDR-AB and non-MDR-AB), the majority (approximately three-fourths) of the patients had a diagnosis of sepsis, with the remaining one-fourth having pneumonia (Table 2). Patients harboring MDR-AB were more likely to have an HCA infection (64.9 % vs. 42.5 %, *p* < 0.001) along with higher illness severity by day 2 of admission (ICU 68.0 % vs. 59.5 %, *p* < 0.001; mechanical ventilation 56.2 % vs. 42.1 %, *p* < 0.001; vasopressors 15.5 % vs. 17.6 %, *p* = 0.420) than non-MDR-AB patients (Table 2). Although patients in the MDR-AB group had a higher prevalence of use of antipseudomonal carbapenems, aminoglycosides, and polymyxins than those in the non-MDR-AB group, they were also far more likely to receive IET (76.2 % MDR-AB vs. 13.8 % non-MDR-AB, *p* < 0.001), regardless of infection type (Fig. 1). Unadjusted hospital mortality among patients with MDR-AB was nearly double that in those with non-MDR-AB (23.7 % vs. 12.7 %, *p* < 0.001).

**Table 2:** Infection characteristics and treatment

	Non-MDR-AB (*n* = 252)	%	MDR-AB (*n* = 1171)	%	*p* Value
Infection characteristics					
Sepsis	184	73.0 %	875	74.7 %	0.573
Pneumonia	68	27.0 %	296	25.3 %	
HCA	107	42.5 %	760	64.9 %	< 0.001
Illness severity measures by day 2					
ICU admission	150	59.5 %	796	68.0 %	0.010
Mechanical ventilation	106	42.1 %	658	56.2 %	< 0.001
Vasopressors	39	15.5 %	206	17.6 %	0.420
Antibiotics administered by day 2					
Antipseudomonal penicillins with β-lactamase inhibitor	140	55.6 %	588	50.2 %	0.124
Extended-spectrum cephalosporins	100	39.7 %	373	31.9 %	0.017
Antipseudomonal fluoroquinolones	96	38.1 %	489	41.8 %	0.284
Antipseudomonal carbapenems	37	14.7 %	350	29.9 %	< 0.001
Aminoglycosides	25	9.9 %	204	17.4 %	0.003
Penicillins with β-lactamase inhibitors	4	1.6 %	19	1.6 %	1.000
Tetracyclines	3	1.2 %	6	0.5 %	0.203
Folate pathway inhibitors	3	1.2 %	11	0.9 %	0.724
Polymyxins	0	0.0 %	37	3.2 %	0.001
Empiric treatment appropriateness					
Non-IET	162	64.3 %	217	18.5 %	< 0.001
IET	26	10.3 %	693	59.2 %	
Indeterminate	64	25.4 %	261	22.3 %	

*Abbreviations: MDR-AB* multidrug-resistant *Acinetobacter baumannii*, *HCA* healthcare-associated, *ICU* intensive care unit, *IET* inappropriate empiric therapy

**Fig. 2:**
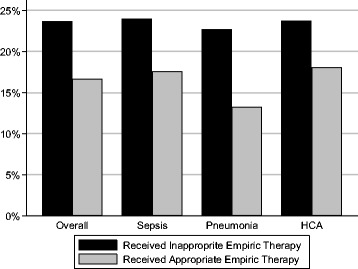
Mortality and inappropriate empiric therapy. *HCA* healthcare-associated

When we compared the cohort of 1098 patients (77.2 % of all AB patients) with valid, known antimicrobial treatment data based on the receipt of IET, we found that only 379 (34.5 %) received appropriate therapy (Table 3). The rate of sepsis upon admission did not significantly differ between IET and non-IET patients (Table 3). Unadjusted hospital mortality was higher in patients receiving IET than non-IET (23.6 % vs. 16.6 %, *p* = 0.007) in all infection types (Fig. 2).

**Table 3:** Characteristics of the cohort, based on receipt of inappropriate empiric therapy

	Non-IET (*n* = 379)	%	IET (*n* = 719)	%	*p* Value
Baseline characteristics					
Mean age, years (SD)	62.4 (15.6)		62.7 (15.9)		0.767
Male sex	202	53.3 %	373	51.9 %	0.654
Race/ethnicity					
White	236	62.3 %	464	64.5 %	0.055
Black	103	27.2 %	159	22.1 %	
Hispanic	7	1.8 %	32	4.5 %	
Other	33	8.7 %	64	8.9 %	
Admission source					
Non-healthcare facility (including from home)	223	58.8 %	357	49.7 %	0.022
Clinic	14	3.7 %	15	2.1 %	
Transfer from ECF	69	18.2 %	173	24.1 %	
Transfer from another non-acute care facility	8	2.1 %	20	2.8 %	
Emergency department	63	16.6 %	148	20.6 %	
Other	2	0.5 %	6	0.9 %	
Elixhauser comorbidities					
Congestive heart failure	97	21.5 %	209	30.4 %	0.222
Valvular disease	30	6.6 %	53	7.7 %	0.746
Pulmonary circulation disease	26	5.8 %	62	9.0 %	0.306
Peripheral vascular disease	51	11.3 %	82	11.9 %	0.322
Paralysis	85	18.8 %	176	25.6 %	0.448
Other neurological disorders	82	18.1 %	185	26.9 %	0.133
Chronic pulmonary disease	164	36.3 %	305	44.4 %	0.786
Diabetes without chronic complications	105	23.2 %	241	35.1 %	0.049
Diabetes with chronic complications	38	8.4 %	56	8.2 %	0.208
Hypothyroidism	49	10.8 %	119	17.3 %	0.113
Renal failure	107	23.7 %	217	31.6 %	0.501
Liver disease	17	3.8 %	27	3.9 %	0.557
Peptic ulcer disease with bleeding	0	0.0 %	0	0.0 %	1.000
AIDS	0	0.0 %	0	0.0 %	1.000
Lymphoma	2	0.4 %	10	1.5 %	0.236
Metastatic cancer	24	5.3 %	17	2.5 %	0.001
Solid tumor without metastasis	18	4.0 %	19	2.8 %	0.066
Rheumatoid arthritis/collagen vascular	11	2.4 %	29	4.2 %	0.342
Coagulopathy	58	12.8 %	83	12.1 %	0.077
Obesity	48	10.6 %	128	18.6 %	0.027
Weight loss	94	20.8 %	250	36.4 %	0.001
Fluid and electrolyte disorders	219	48.5 %	393	57.2 %	0.322
Chronic blood loss anemia	5	1.1 %	9	1.3 %	0.924
Deficiency anemia	173	38.3 %	363	52.8 %	0.127
Alcohol abuse	18	4.0 %	24	3.5 %	0.246
Drug abuse	16	3.5 %	19	2.8 %	0.157
Psychosis	22	4.9 %	45	6.6 %	0.765
Depression	50	11.1 %	96	14.0 %	0.941
Hypertension	215	47.6 %	413	60.1 %	0.821
Charlson comorbidity score					
0	72	19.0 %	164	22.8 %	0.152
1	108	28.5 %	177	24.6 %	
2	64	16.9 %	151	21.0 %	
3	62	16.4 %	108	15.0 %	
4	34	9.0 %	66	9.2 %	
5+	39	10.3 %	53	7.4 %	
Mean (SD)	2.2 (2.2)		2.0 (1.9)		0.043
Median [IQR]	2 [1–3]		2 [1–3]		0.202
Infection characteristics and treatments					
Infection characteristics					
Sepsis	296	78.1 %	525	73.0 %	0.065
Pneumonia	83	21.9 %	194	27.0 %	
HCA	222	58.6 %	464	64.5 %	0.053
MDR-AB	217	57.3 %	693	96.4 %	< 0.001
Illness severity					
ICU admission	249	65.7 %	482	67.0 %	0.655
Mechanical ventilation	206	54.4 %	390	54.2 %	0.972
Vasopressors	64	16.9 %	121	16.8 %	0.981
Antibiotics administered					
Antipseudomonal penicillins with β-lactamase inhibitor	91	24.0 %	123	17.1 %	0.006
Antipseudomonal fluoroquinolones	97	25.6 %	209	29.1 %	0.222
Extended-spectrum cephalosporins	177	46.7 %	339	47.1 %	0.888
Antipseudomonal carbapenems	190	50.1 %	350	48.7 %	0.647
Aminoglycosides	140	36.9 %	269	37.4 %	0.877
Penicillins with β-lactamase inhibitors	5	1.3 %	9	1.3 %	0.924
Polymyxins	12	3.2 %	9	1.3 %	0.028
Folate pathway inhibitors	7	1.8 %	23	3.2 %	0.191
Tetracyclines	1	0.3 %	4	0.6 %	0.665
Hospital characteristics					
U.S. region					
Midwest	118	31.1 %	234	32.5 %	< 0.001
Northeast	60	15.8 %	91	12.7 %	
South	167	44.1 %	254	35.3 %	
West	34	9.0 %	140	19.5 %	
Number of beds					
< 200	41	10.8 %	78	10.8 %	0.011
200–299	65	17.2 %	165	22.9 %	
300–499	143	37.7 %	292	40.6 %	
500+	130	34.3 %	184	25.6 %	
Teaching	212	55.9 %	292	40.6 %	< 0.001
Urban	357	94.2 %	696	96.8 %	0.038
Hospital mortality	63	16.6 %	170	23.6 %	0.007

*Abbreviations: IET* inappropriate empiric therapy, *ECF* extended care facility, *HCA* healthcare-associated, *MDR-AB* multidrug-resistant *Acinetobacter baumannii*, *ICU* intensive care unit

In a regression model designed to explore the impact of MDR on the risk of IET exposure, MDR-AB was the single strongest predictor of receiving IET (adjusted relative risk ratio 5.5, 95 % CI 4.0–7.7, *p* < 0.001) (Table 4). The confirmatory analyses produced similar risk ratios (Table 4).

**Table 4:** Adjusted risk of inappropriate empiric therapy and hospital mortality

Risk of IET in the setting of MDR-AB	Marginal effect, IET in non-MDR-AB	Marginal effect, IET in MDR-AB	Adjusted relative risk ratio (95 % CI)	*p* Value
Method				
Parse model	13.8 %	76.2 %	5.5 (4.0–7.7)	< 0.001
Propensity score (based on 204 matched pairs; 81.0 % matched)	13.4 %	73.9 %	5.5 (3.6–8.4)	< 0.001
Nonparse model	14.4 %	75.6 %	5.3 (3.7–7.4)	< 0.001
Risk of death in the setting of IET	Marginal effect, mortality in non-IET	Marginal effect, mortality in IET	Adjusted relative risk ratio (95 % CI)	*p* Value
Method				
Parse model	15.9 %	24.3 %	1.53 (1.21–1.93)	< 0.001
Propensity score (based on 226 matched pairs; 59.6 % matched)	15.0 %	27.8 %	1.85 (1.35–2.54)	< 0.001
Nonparse model	14.5 %	25.6 %	1.76 (1.36–2.28)	< 0.001

*IET* inappropriate empiric therapy, *MDR-AB* multidrug-resistant *Acinetobacter baumannii*

In a nonparse generalized regression model adjusting for all confounders (demographics, comorbidities, severity of illness measures, hospital characteristics), IET was associated with an increased risk of in-hospital mortality (adjusted relative risk ratio 1.76; 95 % CI 1.36–2.28, *p* < 0.001 (Table 4). The parse model and propensity-matched analysis produced similar risk ratios.

## Discussion

In this large, multicenter cohort study, we have demonstrated that CA and HCA pneumonia and sepsis are rarely caused by AB. However, when AB is present, it is most often MDR. Moreover, harboring MDR puts patients at a fivefold increased risk of receiving IET, which is in turn associated with increased hospital mortality.

Multiple investigators have documented the exceedingly high and rising rate of AB resistance. In a multicenter microbiology database study in the United States, we noted a rise in MDR-AB from 21.4 % between 2003 and 2005 to 35.2 % in the 2009–2012 period [3]. Similarly, the Center for Disease Dynamics, Economics & Policy (CDDEP) reported an MDR-AB increase from 32.1 % in 2009 to 51.0 % in 2010 [2]. The discrepancy between the two studies reflects the populations evaluated and the definitions of MDR applied. While our prior investigation was limited to only patients with severe sepsis and septic shock, the CDDEP surveillance included all infection sources. Additionally, we limited drug definitions to those where clinical efficacy data were available, while CDDEP included all pertinent drug categories.

Our present study, though not longitudinal, confirms the high probability of MDR-AB, though the rate is higher than that in either of the surveillance studies. Although the our examined population is more similar to that in our previous surveillance study than to the CDDEP surveillance, the IET definition is more in line with that of the CDDEP [19]. Because our data represent years 2009–2013, the high prevalence may simply be consistent with continued growth of this resistant pathogen beyond the time frame examined in either of the previous surveillance efforts.

We confirm that antimicrobial resistance confers a high risk for IET. A previous single-center study reported that having severe sepsis or septic shock caused by carbapenem-resistant AB doubled the risk of IET [14]. This is the case for any gram-negative pathogen of severe sepsis or septic shock [24]. In the present study, the effect size was even greater, with a more than fivefold increase in the relative risk of receiving IET compared with non-MDR-AB. This suggests that clinicians should consider broad empiric coverage when AB is either suspected or identified by rapid testing.

In sepsis and pneumonia, it has been shown repeatedly that IET increases hospital mortality two- to fourfold and that escalation of treatment in response to culture results fails to alter this outcome [4–13]. Specific to AB sepsis and septic shock patients, Shorr et al. recently reported a significantly elevated risk of mortality associated with IET (risk ratio 1.42, 95 % CI 1.10–1.58, *p* = 0.015) [14]. We confirm this observation in a cohort of patients with AB pneumonia or sepsis. However, this association has not always been found in studies of AB infection. While researchers in two additional cohort studies reported a two- to sixfold rise in hospital mortality in association with IET for AB, six other study groups failed to detect such an association [25–32]. Though it is not clear why such a well-recognized relationship would not exist specifically in the setting of AB, there are a number of potential reasons for this divergence. Some of the previous studies suffer from several methodological issues, such as small sample size, incomplete adjustment for or unmeasured confounders, and overadjusting for some factors that may be collinear.

Our study has a number of strengths and limitations. It included a large multicenter cohort representative of U.S. institutions and thus has broad generalizability. Though largely representative of U.S. institutions overall, the southern portion of the United States is overrepresented in the database. Although this made the study susceptible to bias, particularly selection bias, we dealt with it by setting a priori enrollment criteria and definitions for the main exposures and outcomes. Though some misclassification is possible, the main exposures (MDR-AB, IET) and outcomes (IET, hospital mortality) are minimally susceptible to misclassification. At the same time, in at least some of the identified cases, AB might have represented colonization rather than true infection. Additionally, the fact that fully one-third of all MDR-AB were isolates from cases defined as CA suggests that some misclassification may exist in this group; that is, it is possible that we were unable to identify these patients’ exposure to the healthcare system with the variables available in the current database. Although confounding is a potential issue in observational studies, we attempted to eliminate this through regression analyses using a large number of potentially confounding variables. Nevertheless, the possibility of residual confounding remains.

## Conclusions

In this largest representative multicenter study to date, although AB was a rare pathogen in CA or HCA pneumonia or sepsis, over 80 % of the AB isolates exhibited MDR. MDR increased the risk of receiving IET fivefold. In turn, IET was associated with increased risk of in-hospital mortality.

## Key messages

- AB is a rare pathogen in community-acquired or healthcare-associated pneumonia or sepsis.
- Eighty percent of all AB in this population is MDR.
- MDR raises the risk of receiving inappropriate empiric therapy fivefold.
- Inappropriate empiric therapy increases the risk of hospital mortality.

## Abbreviations

AB, *Acinetobacter baumannii*; CA, community-acquired; CDDEP, Center for Disease Dynamics, Economics & Policy; ECF, extended care facility; HCA, healthcare-associated; I, intermediate; ICU, intensive care unit; IET, inappropriate empiric therapy; IRB, institutional review board; MDR, multidrug resistance; PDR, pandrug-resistant; R, resistant; RRR, relative risk ratio; S, susceptible; XDR, extensively drug resistant
